# Exercises in Practical Physiology

**Published:** 1905-09

**Authors:** 


**Exercises in Practical Physiology.** By AUGUSTUS D. WALLER, M.D., F.R.S. Part II.—*Exercises and Demonstrations in Chemical and Physical Physiology*, by Augustus D. Waller and W. Legge Symes. Pp. iv., 79. London: Longmans, Green & Co. 1905.—This little book seems to us an exceedingly good one on that branch of practical physiology of which it treats, and admirably adapted to the purpose for which it is intended. Although not lengthy, it contains all the matter which it is important for a medical student to know; and one who has carefully worked through this course under his teacher's supervision, and remembers the facts he has here learnt by practical experience, will have a knowledge of this important part of physiology which will always be useful to him. The composition and arrangement of the book show that its authors have had a large experience of teaching, and are thoroughly conversant with the needs of the student. We can cordially recommend it.

